# Radiographic response to neoadjuvant therapy and its impact on scope of surgery and prognosis in stage IIB/III soft tissue sarcomas

**DOI:** 10.1186/1471-2407-13-591

**Published:** 2013-12-11

**Authors:** Yong Chen, Yun Yang, ChunMeng Wang, YingQiang Shi

**Affiliations:** 1 Department of Gastric Cancer and Soft Tissue Tumors, Fudan University Shanghai Cancer Center, Shanghai 200032, China; 2 Department of Oncology, Shanghai Medical College, Fudan University, Shanghai 200032, China; 3 Department of Bone and Soft Tissue Tumors, Tianjin Medical University Cancer Institute and Hospital, Tianjin 300060, China

## Abstract

**Background:**

Neoadjuvant chemoradiotherapy has been newly included in the NCCN guidelines as a treatment option for stage IIB/III soft tissue sarcomas. Whether radiographic response to neoadjuvant therapy correlates with improved quality of resection and prognosis remains unproven.

**Methods:**

Data from 120 consecutive patients who were treated with neoadjuvant chemoradiotherapy followed by surgical resection for their locally aggressive limb sarcomas were retrospectively reviewed. Radiographic response was evaluated after neoadjuvant therapy according to Response Evaluation Criteria In Solid Tumors, and data was analyzed for overall survival (OS), local recurrence free survival (LRFS) and metastasis free survival (MFS). Surgical complications and toxicities, as well as functional outcomes, were also analysed.

**Results:**

After neoadjuvant chemoradiotherapy, 25 patients (20.8%) had a partial response, 75 patients (62.5%) had stable disease, and 20 patients (16.7%) showed disease progression. Radiographic response to neoadjuvant therapy correlated significantly with improved OS (P = 0.002) and MFS (P < 0.001). Patients with partial response (PR) had a significantly decreased rate of R2 resection as compared with stable disease (SD) and progressive disease (PD) patients (4.0% Vs 21.4%, P < 0.001).

**Conclusions:**

Radiographic response to neoadjuvant chemoradiotherapy correlates with improved quality of resection and prognosis in extremity STS patients.

## Background

Soft tissue sarcomas (STS) account for approximately 1% of all adult malignancies [1]. At the time of primary diagnosis, about 20% of patients have stage IIB/III disease, as evaluated by American Joint Committee on Cancer (AJCC) [2]. These tumors are high-grade, >5 cm in size, deep to investing fascia and are considered high-risk with a 5-year survival rate of approximately 50% [3]. As reported previously, about 42% of patients with stage IIB/III STS have extensive or locally advanced sarcomas, which makes resection with safe margins and satisfactory functional outcomes very challenging [4]. Treatment modalities for these patients, as recommended by current National Comprehensive Cancer Network (NCCN) guidelines, comprise a multidisciplinary approach of preoperative chemotherapy and/or radiotherapy (RT) followed by surgery and adjuvant chemoradiotherapy [5].

Preoperative treatment is now widely used in many cancers, including STS. Neoadjuvant chemotherapy has been shown to correlate with improved disease free survival and overall survival in STS patients [6,7], and preoperative radiation has been shown to reduce tumor burden before resection, allowing more conservative or function-sparing surgeries [8]. Radiographic response has been introduced as a useful measure to evaluate tumor response to preoperative treatment, and has been correlated with improved local control and overall survival in a small cohort study [9]. However, whether radiographic response to neoadjuvant chemotherapy in high risk STS patients translates into a reduction in the scope of surgical resection, which would facilitate limb-salvage operations, remains unanswered. Additionally, the toxicities, surgical complications, and functional outcomes associated with multimodal treatment require further investigation.

In this study, we aim to answer whether response to neoadjuvant therapies correlated wth decreased scope of surgery and decreased rate of R2 resection in locally aggressive STS. Treatment-related toxicities, functional outcomes and surgical complications were also reviewed. We further investigate whether radiographic response to neoadjuvant therapy correlates significantly with improved overall survival (OS), metastasis free survival (MFS) and resection quality.

## Methods

### Patient population

One hundred twenty consecutive patients with locally aggressive extremity STS of AJCC stage IIB/III who were treated with neoadjuvant therapy protocol at the TianJin Medical University Cancer Institute and Hospital (TJCIH) from 1993 to 2009 represented the study population, all patients had at least one vital structures of the following involved (Encased, partially encircled or infiltrated) by tumor on presentation: major vessels, vital nerves and long tubular bones. The study was approved by institutional review board of TJCIH. Tumor size was defined as the maximum diameter recorded upon presentation using cross-sectional imaging. Tumors located in the upper extremities (n = 33) including shoulder (n = 12) and axilla (n = 10), and lower extremities (n = 87) including groin (n = 9), hip (n = 11) and buttock (n = 14) were included. General pathologic features recorded included tumor size, French Federation of Cancer Centres (FNCLCC) grade [10], histologic subtype [11] and microscopic margins [12].

### Neoadjuvant therapy protocol

The neoadjuvant therapy protocol was administered in a nonrandomized fashion. All patients had computed tomography (CT) or magnetic resonance imaging (MRI) studies of their primary lesion before and after neoadjuvant therapy. All CT scans were performed with intravenous contrast enhancement. All MRI studies were performed with standardized T1 and T2 weighting. The neoadjuvant therapy protocol was systemic chemotherapy using a MAID (Mesna + Adriamycin + Ifosfomide + Dacarbazine) or AIM (Adriamycin + Ifosfomide + Mesna) regimen for 1 to 2 cycles (Dosage in both regimens: Ifosfomide: 2 g/m^2^ × 5 days; Adriamycin: 30 mg/m^2^ × 2 days; Dacarbazine: 400 mg/m^2^ × 5 days and Mesna: 2 g/m^2^ × 5 days). Neoadjuvant chemotherapy regimen choice and duration were at the discretion of the treating surgical oncologist based on patient condition, response to first cycle, or patient decision after cycle 1. Three weeks after completion of neoadjuvant chemotherapy, radiographic response was assessed by CT or MRI using the Response Evaluation Criteria in Solid Tumors (RECIST) criteria, and reassessment of resectability was done in accordance with methodology used by Meric et al. [9]. Reassessment of tumor size was performed by radiologists blinded to clinical characteristics, and reassessment of surgical scope was performed by the surgical oncologist tasked to perform the resection.

Patients with PR (decrease in scope of surgery) were assigned to surgical resection without neoadjuvant RT. Patients with SD (no change in scope of surgery) and patients with PD (increase in scope of surgery) were assigned to neoadjuvant RT at dosage of 50Gy (2Gy fraction per day, 5 days per week for 5 successive weeks), given until completion, patient refusal, or unacceptable RT-related toxicities. Neoadjuvant RT was followed by the same imaging studies, reassessment and operations as for all other patients.

The National Cancer Institute Common Toxicity Criteria, version 3.0, were used to assess chemotherapy-related toxicity and the Radiation Therapy Oncology Group (RTOG) acute and chronic toxicity criteria were used to describe toxicity due to RT [13].

### Surgery and post-operative treatment

All one hundred and twenty patients underwent surgical resection 3 to 4 weeks after completion of neoadjuvant therapy. A decreased scope of surgery was acknowledged by multidisciplinary team that one or more vital structure could be spared or more normal tissue could be preserved (PR) after neoadjuvant therapies. In patients with tumor response (PR) to neoadjuvant therapies, limb salvage surgical resection was performed per normal clinical practice through grossly normal tissues. In patients wth SD after neoadjuvant therapies, limb-salvage surgical resection with reconstruction was perfomed as following: If the tumor was found during surgery to abut a major vascular structure, resection and reconstruction of the vasculature were performed with autograft or artificial vessels. If the tumor invaded bone shaft, major bone resection and reconstruction were performed with implantable endoprostheses or plate and screws. If the tumor surrounded or compressed a major nerve, the epineurium was removed in continuity with the tumor and the nerve was treated with anhydrous alcohol for 20 minutes intra-operatively. Moreover, vascular or rotational musculocutaneous flap was applied when necessary. Patients with PD even after neoadjuvant chemo and radiotherapy were assigned to amputation if consenting. Some patients with PD chose limb-salvage operations despite risk of R2 resection.

Post-operative chemotherapy consisted of MAID or AIM at same dosage as preoperative regimens for patients with PR or SD and second line chemotherapy (high dose ifosfamide for synovial sarcomas and gemcitabine + docetaxel for others) for patients with PD after neoadjuvant therapy. For both groups, adjuvant chemotherapy was administered every 3 weeks for 2 cycles, followed by adjuvant radiotherapy, and 2 more cycles of chemotherapy.

Adjuvant radiotherapy was administered to primary tumor site after completion of two cycles of adjuvant chemotherapy, but within first 3 post-operative months for all patients undergoing limb-salvaging surgeries, unless patients refused or demonstrated unacceptable RT-related toxicities. Some patients received RT both before and after surgery, the dosage was based on previous neoadjuvant radiotherapy and should not exceed a total of 6500 cGy per patient.

Post-treatment followup was performed every 3 months for the first 2 years after surgery, then twice annually for 2–3 years, and once annually thereafter. Surgical complications were evaluated as proposed by Daniel et al. according to a 5-level grading system [14]. In brief, the complications were graded according to the treatment modalities and adverse results to the patients. For instance, grade 1 refers to a situation that requires a bedside debridement of wound, grade 2 is an unexpeted bleeding which result in prolonged hospitalization, grade 3 refers to re-operation, grade 4 is perioperative lost of organ or extremity, and grade 5 refers to death. Evaluation of function for the involved extremity was performed using the Functional Evaluation System proposed by Enneking et al. [15], in which patients assessed their pain, function, emotional acceptance, supports, walking, and gait, each on a scale of 0 (worst) to 5 (best) points for a maximum of 30 points.

### Statistics

SPSS 13.0 software was used for statistical analysis. OS, LRFS and MFS were calculated using the Kaplan-Meier method [16]. OS, LRFS, and MFS were defined as the interval from the beginning of treatment to death, to the first local recurrence, and to the first metastasis, respectively. Patients who died from causes unrelated to sarcoma were censored at the time of death. Univariate and multivariate prognostic analyses were performed for OS, LRFS and MFS using the Cox proportional hazards models [17]. The statistically significant variables in the univariate analysis were retained in both multivariate analyses. The conventional 5% significance level was used.

## Results

### Clinical characteristics, pathologic features and treatment modalities

Clinical, pathologic, and treatment variables, and their correlation with radiographic response for all 120 patients are listed in Table 1. Neoadjuvant chemotherapy resulted in 25 cases of PR, 75 cases of SD and 20 cases of PD. Using the RECIST criteria, none of the 95 patients who underwent neoadjuvant radiotherapy demonstrated a complete response (CR) or PR. All patients with tumor response of SD to neoadjuvant chemotherapy demonstrated SD after neoadjuvant radiotherapy. Of the 20 patients with PD after neoadjuvant chemotherapy, 14 had SD and 6 had PD after neoadjuvant radiotherapy.

**Table 1:** The clinical, pathologic, treatment characteristics in patients with stage III STS and their correlations with radiographic response

**Issues**	**Catogaries**	**N**	**Radiographic response**			**χ**	**P**
			**PR**	**SD**	**PD**		
Age	Median	42 years					
Gender	Male	67	15	39	13	−0.02	0.831
	Female	53	10	36	7		
Size(CM)	Mean	10 cm					
	≥10		5	39	19	0.453	<0.001
	<10		20	36	1		
FNCLCC grade	2	45	10	31	4	3.150	0.207
	3	75	15	44	16		
Subtype	MFH	33	6	17	10	11.270	0.187
	SS	28	5	20	3		
	LS	28	8	16	4		
	LMS	16	1	13	2		
	Others	15	5	9	1		
Neo-AC cycles	1	18	7	10	3	0.832	0.374
	2	102	18	65	17		
Chemotherapy regimen	MAID	80	18	50	12	0.720	0.698
	AIM	40	7	25	8		
UICC margin	R0 + R1	92	24	57	11	10.491	0.005
	R2	28	1	18	9		
Follow-up(M)	Median	46.0 (13–158)					

PR: Partial response; SD: Stable Disease; PD: Progressive Disease; FNCLCC: French Federation of Cancer Centres; Neo-AC: Neoadjuvant chemotherapy; UICC: Union Internationale Contre Cancer; MFH: malignant fibrous histiocytoma; SS: synovial sarcoma; LS: liposarcoma; LMS: leiomyosarcoma.

**Table 2:** Univariate and multivariate analysis of variable factors for 5-year OS

**Factors**	**OS**	**Univariate analysis**			**Multivariate analysis**		
		**RR**	**95% CI**	**P**	**RR**	**95% CI**	**P**
Radiographic response				<0.001			
PR (n = 25)	96.0	0.081	0.027-0.239		0.133	0.037-0.483	0.002
SD + PD (n = 95)	38.8	1			1		
Size (cm)				<0.001			0.568
≥10 (n = 63)	33.5	5.276	2.704-10.294		1.263	0.566-2.818	
<10 (n = 57)	81.4	1			1		
FNCLCC Grade				<0.001			0.001
Grade 2 (n = 45)	86.2	1			1		
Grade 3 (n = 75)	38.7	6.188	2.647-14.467		5.146	2.028-13.058	
UICC margins				<0.001			0.005
R0 + R1 (n = 92)	69.9	1			1		
R2 (n = 28)	6.6	8.196	4.604-14.591		2.384	1.301-4.367	
Surgery type				<0.001			0.027
Resection only (n = 55)	89.4	1			1		
Resection with other modalities^*^ (n = 65)	26.9	9.708	4.479-21.042		2.986	1.133-7.865	

^*^: Other modalities include epineural dissection and reconstruction of blood vessels and bony structures. FNCLCC: French Federation of Cancer Centres; UICC: Union Internationale Contre Cancer.

**Table 3:** Univariate and multivariate analysis of variable factors for 5-year local recurrence free survival (LRFS)

**Factors**	**LRFS**	**Univariate analysis**			**Multivariate analysis**		
		**RR**	**95% CI**	**P**	**RR**	**95% CI**	**P**
Radiographic response				0.024			0.351
PR (n = 25)	96.0	0.100	0.014-0.737		0.366	0.044-3.027	
SD + PD (n = 95)	65.0	1			1		
Size (cm)				<0.001			0.572
≥10 (n = 63)	33.3	6.614	2.293-19.079		1.491	0.373-5.950	
<10 (n = 57)	81.4	1			1		
FNCLCC Grade				<0.001			0.120
Grade 2 (n = 45)	86.2	1			1		
Grade 3 (n = 75)	38.6	9.178	2.177-38.697		3.278	0.734-14.649	
UICC margins				<0.001			<0.001
R0 + R1 (n = 92)	69.9	1			1		
R2 (n = 28)	6.6	22.975	9.204-57.353		10.458	3.755-29.126	
Surgery type				<0.001			0.672
Resection only (n = 55)	89.4	1			1		
Resection with other modalities^*^ (n = 65)	27.1	9.184	2.767-30.476		1.431	0.273-7.498	

^*^: Other modalities include epineural dissection and reconstruction of blood vessels and bony structures. FNCLCC: French Federation of Cancer Centres; UICC: Union Internationale Contre Cancer.

**Table 4:** Univariate and multivariate analysis of variable factors for 5-year metastasis free survival (MFS)

**Factors**	**MFS**	**Univariate analysis**			**Multivariate analysis**		
		**RR**	**95% CI**	**P**	**RR**	**95% CI**	**P**
Radiographic response				<0.001			<0.001
PR (n = 25)	96.0	0.072	0.025-0.209		0.098	0.030-0.317	
SD + PD (n = 95)	30.9	1			1		
Size (cm)				<0.001			0.991
≥10 (n = 63)	29.0	3.098	1.821-5.271		1.004	0.519-1.940	
<10 (n = 57)	67.9	1			1		
FNCLCC Grade				<0.001			0.008
Grade 2 (n = 45)	70.2	1			1		
Grade 3 (n = 75)	33.9	2.938	1.632-5.291		2.391	1.255-4.553	
UICC margins				<0.001			0.001
R0 + R1 (n = 92)	60.6	1			1		
R2 (n = 28)	3.6	5.995	3.600-9.983		2.553	1.461-4.462	
Surgery type				<0.001			0.181
Resection only (n = 55)	75.8	1			1		
Resection with other modalities^*^ (n = 65)	22.8	4.882	2.725-8.744		1.638	0.795-3.376	

^*^: Other modalities include epineural dissection and reconstruction of blood vessels and bony structures. FNCLCC: French Federation of Cancer Centres; UICC: Union Internationale Contre Cancer.

On presentation, all 120 patients were evaluated to have amputation as only choice (n = 30) or have to undergo vessel replacement (n = 79) and bony reconstruction (n = 11), 30 patients of the 79 were evaluated to be candidates of epineural resection (n = 30). All 120 patients underwent surgical resection 3 to 4 weeks after completion of neoadjuvant therapy. In patients with tumor response of PR or SD, limb salvage surgical resection was performed (n = 100/120). Patients with PD (n = 20/120) were recommended to undergo amputation because of extensive tumor growth and neurovascular bundle invasion, but only 11/20 patients accepted, while 9/20 patients with PD chose limb-salvage operations despite the risk of R2 resection. In total 109 patients underwent limb salvage resections, 55/109 (50.6%) underwent resection without reconstruction, 54/109 (49.4%) had resection with other modalities such as autograft or artificial vessel replacement due to resection of major vessels (n = 33), inner fixation due to resection of bony structures (n = 11) or epineural dissection and anhydrous alcohol implication due to proximity of major nerves to tumor (n = 20).

All 120 patients were treated with adjuvant chemotherapy according to hospital protocol. Gemcitabine + docetaxel was administered to 17 patients while 3 patients received high dose ifosfamide. In all 109 patients who underwent limb-salvage operations, 95 patients had radiotherapy both before and after surgery, with a total dose of 6500 cGy, 14 patients underwent post-operative external beam radiotherapy only (400 cGy fraction per day, 5 days per week for successive 4 weeks) with a total dose of 6500 cGy.

### Complications and toxicities

There was no treatment associated death in this study. One patient had a grade IV surgical complication of acute arterial embolization in the lower femoral artery by the second post-operative day and was treated with above knee amputation. Nine patients (9/120, 7.5%) had grade III surgical complications and had re-operations. Grade II and grade I surgical complications were seen in 20 (16.7%) and 22 (18.3%) patients respectively, with most of the cases well managed with additional antibiotics and debridements at the bedside. Altogether, surgical complications were seen in 52 patients (43.3%) in our study, most of these were mild to moderate, indicating that our aggressive treatment protocol was feasible and safe.

Forty-one out of 120 patients (34.2%) in total experienced grade 4 toxicities due to chemo- or radiotherapy; thirth-four patients (28.3%) experienced grade 4 hematologic toxicities, and ten patients (8.3%) experienced grade 4 nonhematologic toxicities. Chemo-associated toxicities included grade 4 leukopenia in twenty-seven, grade 4 thrombocytopenia in twelve, grade 4 anemia in six, grade 4 liver function toxicities in two and grade 4 nausea and vomiting in two. Radio-associated toxicities included grade 4 RT-associated cutaneous ulceration and infection in four and femoral shaft fracture at the site of post-operative radiotherapy in two. Three patients had both grade 4 chemo-associated and radio-associated toxicities.

### Response to neoadjuvant therapy correlated with decreased scope of surgery in locally aggressive extremity STS patients with acceptable functional outcomes

We first investigated whether response to neoadjuvant chemoradiotherapy correlated with decreased scope of surgery in patients with extensive or locally advanced sarcomas. After neoadjuvant therapy, 20/120 patients who had PD were recommended to undergo ablative procedures, of which 11 agreed and 9 chose to undergo limb-salvage operations. In total 109 patients underwent a limb-salvaging operation. Though 28 (28/109, 25.7%) of these limb-salvage patients had a R2 resection, the tumor regression in the 25 patients (25/120, 20.8%) with PR to neoadjuvant chemotherapy translated into decrease in scope of surgery. More precisely, in the 30 patients who were evaluated on presentation to have only amputation as treatment choice, 12 of them had PD and were recommended amputation (8 accepted and 4 refused), 12 others had SD and underwent tumor resection with reconstruction (vessel replacement in 6, epineural resection in 4 and both in 2), the remaining 6 patients had PR and underwent tumor resection without sacrifice of vital structures. Furthermore, in the 79 patients who had vascular involvement on presentation which surgeons would recommend vascular resection, all those presented PR (n = 13) and 33 of 58 who presented SD (33/58, 56.9%) to neoadjuvant therapies had their vessels spared. The above findings indicated that neoadjuvant chemotherapy might play a role in limb-salvage treatment in patients with locally advanced sarcomas. On the other hand, the 75 SD patients who had their diseases evaluated before neoadjuvant therapies to be unresectable were treated with limb-salvage operation, with 18 of the 75 (18/75, 24%) were R2 resection.

We further evaluated post-operative function in limb-salvage patients with MSTS scoring system. The mean score was 26 (87% score, range 16–28), indicating good functional outcomes in these patients, especially considering their poor prognosis on presentation.

### Radiographic response to neoadjuvant therapy is of prognostic value in patients with stage IIB/III STS

**Figure 1:**
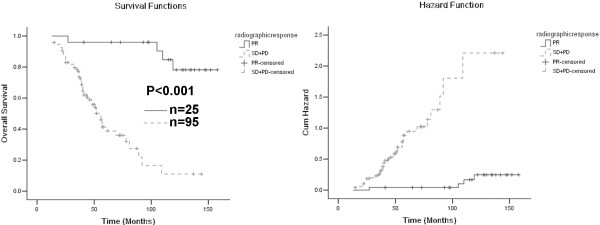
**Left panel: Kaplan-Meier curves of overall survival (OS) according to radiographic response.** Right panel: cumulative incidence curves of risk of death.

**Figure 2:**
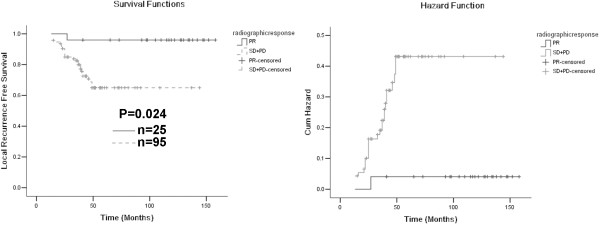
**Left panel: Kaplan-Meier curves of local recurrence free survival (LRFS) according to radiographic response.** Right panel: cumulative incidence curves of risk of local recurrence.

**Figure 3:**
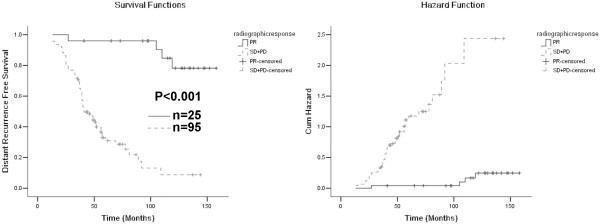
**Left panel: Kaplan-Meier curves of metastasis free survival (MFS) according to radiographic response.** Right panel: cumulative incidence curves of risk of metastasis.

We hypothesized that any decrease in tumor size after neoadjuvant therapy would favorably affect prognosis. Therefore, patients who had radiographic PR were compared with patients who had SD and PD. In univariate analysis, radiographic response correlated with improved OS (Figure 1, Table 2), LRFS (Figure 2, Table 3) and MFS (Figure 3, Table 4). In multivariate analysis, response of PR correlated significantly with OS (HR, 0.133; 95% CI, 0.037-0.483; P = 0.002) and MFS (HR, 0.098; 95% CI, 0.030-0.317; P < 0.001). Patients with PR also had improved LRFS (HR, 0.366; 95% CI, 0.044-3.027) compared with those with SD and PD, but the difference did not reach statistical significance (P = 0.351).

Furthermore, we investigated the impact of radiographic response on resection margin. In the 109 patients who underwent limb-salvaging procedures, resection margin was R2 in 28 patients. For patients with PR (n = 25), SD (n = 75) or PD (n = 9), resection margin was determined to be R2 in 1, 18 and 9 patients (P < 0.001) respectively, indicating that radiographic response correlated with improved quality of resection in these patients.

## Discussion

The goal of surgical therapy for soft tissue sarcoma is to achieve grossly and microscopically negative (R0) margins of resection with the best possible functional results. Our study retrospectively reviewed full data of 120 consecutive cases who had locally advanced sarcomas and were treated with neoadjvuant therapy and surgical resection. We demonstrated that 109 (90.8%) patients underwent a limb-salvaging procedure after neoadjuvant chemoradiotherapy, while 11 patients had amputation (9.2%, 11/120). Though many patients in this series underwent vascular reconstruction, bony reconstruction or perineural dissection, which were key to limb-salvage, radiographic response to neoadjuvant therapies correlated significantly with increased rate of limb-salvage resections (with acceptable function) and with better resection quality. Survival analysis indicated that radiographic response correlated with improved OS, LRFS and MFS, which was in accordance with a previous report of a smaller cohort of patients [9]. As previous data failed to show that neoadjuvant chemotherapy facilitates limb-salvage operation, our study presented, though retrospective, evidence supporting the use of neoadjuvant therapy in patients who are candidates of amputation.

Scope of surgery is of special concern to surgical oncologists and radiologists because it is the most important determinant of resection quality, reconstruction and scope of adjuvant radiotherapy. Decreased scope of surgery is also associated with better functional outcomes and decreased morbidities [18,19], hence, a decrease in scope of surgery has a positive impact on patients' prognosis. Efforts have been made to obtain tumor shrinkage or compartmental limitation in patients with locally advanced STS. Of the available techniques, isolated limb perfusion (ILP) and neoadjuvant chemoradiotherapy are recommended by the current NCCN guidelines. However, there is no persuasive data to show that ILP has an impact on OS, while neoadjuvant chemoradiotherapy has been proven to improve local control and survival in high risk extremity STS patients even over a long-term followup [7,20].

Radiographic evaluation of tumor response to neoadjuvant therapy provides a non-invasive preoperative modality to predict local outcome and survival. After induction therapy, radiographic examination of the primary tumor is also necessary to create a surgical plan [21]. Recently, the reliability of radiographic response (CT or MRI) as a prognostic tool has been questioned [18], pre-operative positron emission tomography (PET) or post-operative pathologic necrosis were introduced to assess response to pre-operative therapy [22,23], but pathologic necrosis can only be evaluated post-operatively, thus has no predictive value for scope of surgery and functional outcome, while PET is very expensive, evidence-based data for its use in STS is limited [24,25], furthermore, it is of limited help for surgical planning--- surgeons will resect all possible lesions even if PET indicates they are inactive. MRI or CT give the same data as PET for planning surgical scope, but are far cheaper. Thus, the practical feasibility and cost-effectiveness of using CT or MRI vs PET for radiographic evaluation of tumor response to neoadjuvant therapies favors their routine use as a prognostic tool in cancer centers, especially those in developing countries.

Patients with radiographic response of PR or SD to neoadjuvant therapies were all considered to have clinical benefit from treatment according to RECIST criterion. In our study, we found that patients with radiographically-determined PD had poorer prognosis compared to that of patients with SD or PR, this finding may be explained by three factors. First, it is accepted that quality of surgical margins independently predicts local control and survival [26]. It is a challenge for surgical oncologists to obtain a safe margin in limb-salvage operations for patients with stage IIB/III STS, especially those with PD to induction therapies. Nine of the 20 patients who had PD in our series underwent limb-salvage resection, all with a positive margin and poor prognosis. Second, rapidly growing sarcomas with largest diameter more than 10 cm are prone to have intra-operative tumor rupture, which was proven recently to predict early metastasis [4]. In all patients (n = 69) who developed systemic metastases in our study, 19 patients (1, 9 and 9 had PR, SD and PD, respectively) had tumor rupture intra-operatively. These patients developed systemic metastases in a median interval of 5 months (range 2–12), while patients without intra-operative tumor rupture developed metastases in a median interval of 21 months (range 6–98, P < 0.001). Third, response to systemic therapy has been proven effective in extending progression free survival and OS in metastatic STS [27], while neoadjuvant chemotherapy was reported to associate with improved disease free survival and OS [7]. The 20 patients with PD to neoadjuvant chemoradiotherapy presented significantly worse OS, MFS and median post-metastasis survival (17 months vs 22 months, P = 0.002) compared with that in patients with SD or PR. This is in accordance with Delaney’s study [7].

The main limitation of this study is its retrospective nature and a small sample size without comparison group. Furthermore, radiographic response evaluated by CT or MRI according to RECIST criteria was based on tumor size alone, and such measures are unable to show changes within the tumor. Cases with extensive liquefying necrosis as a result of induction therapy, which were evaluated as SD or even PD in our preoperative evaluation, might also facilitate a limb-salvage resection because of decreased tumor capsule tension and peri-capsule edema. Thus, combination with evaluation of tumor necrosis after induction therapy might improve the sensitivity and specificity of using radiographic response as an evaluation tool.

## Conclusions

In locally advanced extremity STS patients, response to neoadjuvant chemo and radiotherapy (21% in our study) might associate with improved survival and quality of surgical resection, but more active agents or regimens are needed for patients who do not have a radiographic response to induction therapies (79% of patients in our study). Developments in molecular biology may help identify which patients will respond best to chemo and radiotherapy and further increase response rates.

## Competing interests

The authors declare that they have no competing interests.

## Authors’ contributions

YC drafted the manuscript, carried out the conception and design, analysis and interpretation of data. YY participated in the data acquisition and drafted the manuscript. CMW carried out the statistical analysis and interpretation of data. YQS participated in the design of the study and participated in its design and coordination and helped to draft the manuscript. All authors read and approved the final manuscript.

## Pre-publication history

The pre-publication history for this paper can be accessed here:

http://www.biomedcentral.com/1471-2407/13/591/prepub
